# Uncoupling of fatty acid and glucose metabolism in malignant lymphoma: a PET study

**DOI:** 10.1038/sj.bjc.6690386

**Published:** 1999-05-01

**Authors:** J Nuutinen, H Minn, J Bergman, M Haaparanta, U Ruotsalainen, H Laine, J Knuuti

**Affiliations:** Department of Oncology and Radiotherapy, Turku University Central Hospital, PL 52, 20521 Turku, Finland; and Turku PET Centre, Turku University, Finland

**Keywords:** lymphoma, glucose metabolism, FFA, FDG PET

## Abstract

Increased use of glucose through glycolysis is characteristic for neoplastic growth while the significance of serum-free fatty acids for regulation of energy metabolism in cancer is poorly understood. We studied whether serum-free fatty acids (FFA) interfere with glycolytic metabolism of lymphoproliferative neoplasms as assessed with 2-F^18^-fluoro-2-deoxy-D-glucose ([F^18^]FDG) and positron emission tomography (PET). Twelve patients with newly diagnosed non-Hodgkin's lymphoma (*n* = 9) or Hodgkin's disease (*n* = 3) participated in this study before start of oncologic treatment. Each patient underwent two [F^18^]FDG PET studies within 1 week after overnight fast: once during high fasting serum FFA concentrations and once after reduction of serum FFA by administration of acipimox. Acipimox is a nicotinic acid derivative that inhibits lipolysis in peripheral tissues and induces a striking reduction in circulating FFA concentration. In all cases, dynamic PET imaging over the tumour area was performed for 60 min after injection of [F^18^]FDG. Both graphical analysis (rMR_FDG_) and single scan approach (SUV) were used to compare tumour uptake of [F^18^]FDG under high fasting FFA concentrations and after pharmacologically decreased FFA concentrations. Serum FFA concentrations were reduced significantly from 0.92 ± 0.42 mmol l^−1^ at baseline to 0.26 ± 0.31 mmol l^−1^ after acipimox administration (*P* = 0.0003). Plasma glucose, serum insulin and lactate concentrations were similar during both approaches. The retention of glucose analogue [F^18^]FDG in tumour was similar between baseline and acipimox studies. Median rMR_FDG_ of a total of 12 involved lymph nodes in 12 patients was 21.9 μmol 100 g^−1^ min^−1^ (range 8.7–82.5) at baseline and 20.1 μmol 100 g^−1^ min^−1^ (range 10.7–81.7) after acipimox. The respective values for median SUV were 7.8 (range 3.6–18.6) and 6.0 (range 4.1–20.2). As expected, [F^18^]FDG uptake in myocardium was clearly enhanced by acipimox due to reduction of circulating FFAs. In conclusion, blood fatty acids appear to have minor significance for [F^18^]FDG uptake in lymphoma. This suggests that glucose utilization is uncoupled of FFA metabolism and indicates that glucose-free fatty acid cycle does not operate in lymphomatous tissue. Glucose appears to be the preferred substrate for energy metabolism in tumours, in spite of the high supply of FFAs in the fasting state. Although acipimox and other anti-lipolytic drugs have potential for treatment of catabolic state induced by cancer, they are not likely to interfere with tumour energy metabolism which is fuelled by glucose. © 1999 Cancer Research Campaign


British Journal of Cancer (1999) **80**(3/4), 513–518
© 1999 Cancer Research Campaign
Article no. bjoc.1998.0386

### Uncoupling of fatty acid and glucose metabolism in malignant lymphoma: a PET study

**J Nuutinen[1,2], H Minn[1,2], J Bergman[2], M Haaparanta[2], U Ruotsalainen[2], H Laine[2] and J Knuuti[2]**

[1]Department of Oncology and Radiotherapy, Turku University Central Hospital, PL 52, 20521 Turku, Finland; and [2]Turku PET Centre, Turku University, Finland

**Summary** Increased use of glucose through glycolysis is characteristic for neoplastic growth while the significance of serum-free fatty acids for regulation of energy metabolism in cancer is poorly understood. We studied whether serum-free fatty acids (FFA) interfere with glycolytic metabolism of lymphoproliferative neoplasms as assessed with 2-$F^{18}$-fluoro-2-deoxy-D-glucose ([$F^{18}$]FDG) and positron emission tomography (PET). Twelve patients with newly diagnosed non-Hodgkin's lymphoma ($n$ = 9) or Hodgkin's disease ($n$ = 3) participated in this study before start of oncologic treatment. Each patient underwent two [$F^{18}$]FDG PET studies within 1 week after overnight fast: once during high fasting serum FFA concentrations and once after reduction of serum FFA by administration of acipimox. Acipimox is a nicotinic acid derivative that inhibits lipolysis in peripheral tissues and induces a striking reduction in circulating FFA concentration. In all cases, dynamic PET imaging over the tumour area was performed for 60 min after injection of [$F^{18}$]FDG. Both graphical analysis ($rMR_{FDG}$) and single scan approach (SUV) were used to compare tumour uptake of [$F^{18}$]FDG under high fasting FFA concentrations and after pharmacologically decreased FFA concentrations. Serum FFA concentrations were reduced significantly from 0.92 ± 0.42 mmol $l^{-1}$ at baseline to 0.26 ± 0.31 mmol $l^{-1}$ after acipimox administration ($P$ = 0.0003). Plasma glucose, serum insulin and lactate concentrations were similar during both approaches. The retention of glucose analogue [$F^{18}$]FDG in tumour was similar between baseline and acipimox studies. Median $rMR_{FDG}$ of a total of 12 involved lymph nodes in 12 patients was 21.9 μmol 100 $g^{-1}$ $min^{-1}$ (range 8.7–82.5) at baseline and 20.1 μmol 100 $g^{-1}$ $min^{-1}$ (range 10.7–81.7) after acipimox. The respective values for median SUV were 7.8 (range 3.6–18.6) and 6.0 (range 4.1–20.2). As expected, [$F^{18}$]FDG uptake in myocardium was clearly enhanced by acipimox due to reduction of circulating FFAs. In conclusion, blood fatty acids appear to have minor significance for [$F^{18}$]FDG uptake in lymphoma. This suggests that glucose utilization is uncoupled of FFA metabolism and indicates that glucose-free fatty acid cycle does not operate in lymphomatous tissue. Glucose appears to be the preferred substrate for energy metabolism in tumours, in spite of the high supply of FFAs in the fasting state. Although acipimox and other anti-lipolytic drugs have potential for treatment of catabolic state induced by cancer, they are not likely to interfere with tumour energy metabolism which is fuelled by glucose.

**Keywords**: lymphoma; glucose metabolism; FFA; FDG PET

Impaired glucose tolerance, insulin resistance, increased nitrogen catabolism and lipolysis are among the main metabolic abnormalities associated with cancer (Tayek, 1992). Glucose is presumably the predominant fuel for tumour energy metabolism, while peripheral glucose consumption of cancer patients is decreased in comparison to healthy weight-matched controls (Minn et al, 1994). The catabolic state of cancer is reflected in increased proteolysis and lipolysis in skeletal muscle and use of the muscle degradation products and lactate for liver gluconeogenesis (Toomey, 1995). The net effect is redistribution of body energy resources in favour of tumour growth. These metabolic changes will ultimately cause cachexia, which is characterized by a network of complex control mechanisms (Kern et al, 1988).

In tumour cells, lipid oxidation is reduced in comparison to normal cells, although labelled fatty acids may show a moderate to high accumulation in neoplastic tissue based on increased phospholipid use for synthesis of cell membranes (Cederbaum et al, 1976; Nariai et al, 1994). As a consequence of the degradation of fat in muscle through increased lipolysis, the free fatty acids (FFA) are ultimately consumed by the host to compensate for increased needs of energy metabolism. It has been hypothesized that this vicious circle of general catabolic state could be interrupted by inhibition of lipolysis. This is the rationale for anti-lipolytic therapy which aims to counteract negative energy balance of cancer cachexia (Obeid et al, 1997). Theoretically, anti-lipolytic drugs should decrease the availability of FFA as a substrate for tumour metabolism, although little is known about the net effect of anti-lipolysis in terms of tumour growth and restoration of host tissues (Mulligan et al, 1992).

The present widespread use of 2-($^{18}$F)-fluoro-2-deoxy-*D*-glucose ([$F^{18}$]FDG) in oncology (Conti et al, 1996) is based on early observations by Warburg (1930) and others (Gullino et al, 1967) indicating increased metabolic demand for glucose in neoplastic tissue. [$F^{18}$]FDG is an analogue of *D*-glucose that competes with glucose for facilitated intracellular transport and phosphorylation by hexokinase (Phelps et al, 1979). The phosphorylated [$F^{18}$]FDG is unable to enter the subsequent metabolic pathways and thus preferentially accumulates in cells with a low phosphatase activity, such as most cancers (Wahl et al, 1993).

*Received 25 June 1998*
*Revised 28 September 1998*
*Accepted 20 October 1998*

*Correspondence to:* J Nuutinen, Department of Oncology and Radiotherapy, Turku University Central Hospital, PL 52, 20521 Turku, Finland

**Table 1** Characteristics of 12 patients with lymphoma studied by FDG PET

| Patient | Age | Sex | Histology (Kiel); grade (WF) | Stage | IPI | Weight kg$^{-1}$ | BMI kg m$^{-2}$ |
|---|---|---|---|---|---|---|---|
| 1 | 39 | M | Centroblastic-centrocytic, folliculare; Low | IVA | 1 | 76 | 25 |
| 2 | 39 | M | Diffuce, mixed; Intermediate | IIB | 0 | 79 | 24 |
| 3 | 37 | M | Centroblastic-centrocytic, folliculare; Low | IIA | 0 | 68 | 22 |
| 4 | 55 | F | Centroblastic-centrocytic, folliculare; Low | IVA | 2 | 67 | 26 |
| 5 | 21 | F | Nodular sclerosis; Hodgkin | IIIA | 2 | 75 | 28 |
| 6 | 50 | M | Mixed cellularity; Hodgkin | IIIBS | 2 | 57 | 20 |
| 7 | 75 | F | Lymphoblastic; High | IVBE | 4 | 62 | 22 |
| 8 | 44 | M | Centroblastic-centrocytic, folliculare; Low | IIIA | 1 | 91 | 26 |
| 9 | 52 | F | Nodular sclerosis; Hodgkin | IIBE | 0 | 59 | 21 |
| 10 | 34 | M | Lymphoblastic; High | IA | 0 | 87 | 24 |
| 11 | 72 | M | Centroblastic-centrocytic; Intermediate | IIIB | 2 | 70 | 23 |
| 12 | 72 | M | Centroblastic-centrocytic; Intermediate | IIA | 1 | 75 | 24 |

F = female, M = male. Staging according to the Ann Arbor classification system: A = no B-symptoms, B = with B-symptoms, E = extranodal, S = splenic. IPI = the international NHL prognostic index. BMI = body mass index.

Based on the simple uptake kinetics of [F[18]]FDG, quantification of glucose utilization in normal tissues is feasible by positron emission tomography (PET) using a three-compartment model (Phelps et al, 1979). [F[18]]FDG PET is also applicable for repeated, non-invasive studies on tumour and host metabolism (Minn et al, 1994; Lapela et al, 1995), although calculation of true glucose metabolic rate is difficult owing to the heterogenous nature of tumour tissue (Spence et al, 1998).

Preliminary evidence suggests that FFA utilization of peripheral tissues is unaffected by tumour which, in turn, preferentially uses glucose in the fasting state (Norton et al, 1980). To our knowledge, it has not been studied whether the close interaction of glucose and fatty acid metabolism seen in skeletal muscle is preserved in cancer tissue. We used [F[18]]FDG and PET to study the in vivo relationship between fatty acid and glucose metabolism in patients with untreated lymphoma. Our aim was to assess how inhibition of lipolysis affects uptake of the glucose analogue [F[18]]FDG in tumour in patients who have fasted overnight.

### MATERIALS AND METHODS

### Patients

Twelve patients with untreated non-Hodgkin's lymphoma (NHL) ($n$ = 9) and Hodgkin's disease ($n$ = 3) admitted to the Turku University Central Hospital, Department of Oncology and Radiotherapy between October 1995 and November 1996 participated in the study. The criteria for eligibility was untreated, histologically verified lymphoproliferative malignancy with at least one evaluable tumour larger than 2 cm in diameter in a non-diabetic, cooperative patient with a World Health Organization performance status better than three. All except two patients had no prior history of cardiac disease. Of the remaining two, one had a mild heart dysfunction and one had hypertension. One of the patients (Table 1, no. 7) had thyroid lymphoma and hypothyroidism (S-TSH 68 mU l$^{-1}$, S-T$_4$-v < 2 pmol l$^{-1}$) at the time of the PET studies.

Eight patients were male and four women. The median age was 47 years (range 21–75). The median body mass index (BMI) in this normal-weighted population was 24 kg m$^{-2}$ (range 20–28) (Olefsky et al, 1991). All patients had complete blood counts and liver function tests that were within normal limits. Chest and abdominal computerized tomography (CT) scans and bone marrow biopsy were also performed as a part of the routine diagnostic work-up. Clinical staging was performed according to the Ann Arbor classification system (Carbone et al, 1971). One of the patients had stage I, four stage II, four stage III and three stage IV lymphoma respectively. Five patients had B-symptoms (weight loss, unexplained fever, night sweats) and four of these five patients had experienced during the last 6 months a median weight loss of 9 kg (range 4–10 kg). The international NHL prognostic index (IPI) was also calculated (Shipp et al, 1993). The characteristics of the patients are shown in Table 1. Each patient gave a written informed consent and the study protocol was approved by the Ethical Committee of the Turku University Central Hospital.

### PET study design

Each patient underwent two [F[18]]FDG PET studies, once in the fasting state and once after administration of acipimox (Figure 1). These studies were randomly performed within 1 week (median 4 days, range 1–8). All studies were performed after a 12-h overnight fast. Patients were also advised to have a low-fat diet on the day before the PET studies.

The baseline PET study was performed without any premedication. In the second study, 250 mg of acipimox (Olbetam®, Farmitalia Carlo Erba, Milan, Italy) was given to patients twice

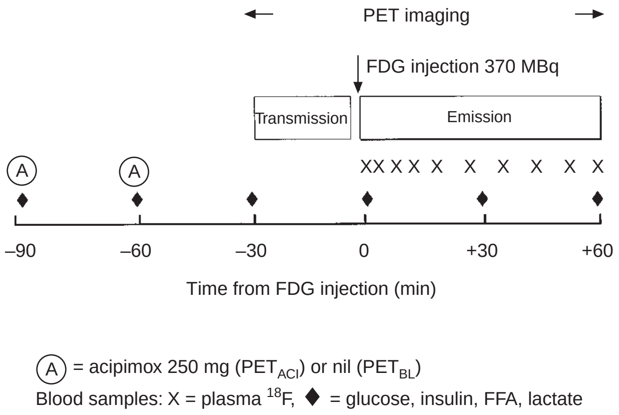

**Figure 1** The design of the PET protocol

 © Cancer Research Campaign 1999

orally 1.5 h and 1 h before [$F^{18}$]FDG injection and imaging. Acipimox is a nicotinic acid derivative that is safely used for treatment of hypercholesterolaemia and hypertriglyceraemia (Nuutila et al, 1994; Knuuti et al, 1995). To prevent the vasodilatory effect of acipimox, seven patients were given a non-steroidal anti-inflammatory agent (mainly ibuprofen) preceding the first acipimox dose (Laverazzi et al, 1989).

### PET imaging

[$F^{18}$]FDG was synthesized as described by Hamacher and co-workers with slight modifications (Hamacher et al, 1986). The radiochemical purity of the tracer was greater than 99%. An ECAT 931/08–12 PET scanner (Siemens/CTI Corp., Knoxville, TN, USA) was used for PET imaging. The device acquires 15 contiguous slices simultaneously with a slice thickness of 6.7 mm; the physical transaxial Full-Width-Half-Maximum in the centre of the field of view is 6.1 mm (Spinks et al, 1988).

To correct for photon attenuation, a transmission scan was obtained prior to emission imaging with a removable ring source containing $^{68}$Ge. A median dose of 363 MBq [$F^{18}$]FDG (range 315–403) was infused with a pump within 2 min into a peripheral or antebrachial vein of the upper extremity. Another venous line was inserted antebrachially in the contralateral pre-heated arm for blood sampling. An emission scan was acquired for 60 min after tracer administration (4 × 30, 3 × 60, 5 × 180, 8 × 300 s). All data were corrected for deadtime, decay and photon attenuation and were reconstructed in a 128 × 128 matrix with a Hann filter (cut-off frequency 0.5). Serial venous blood samples were taken for measurement of radioactivity in plasma over total PET acquisition time. Blood glucose, lactate, FFA and serum insulin were determined before, midway and after the PET study.

### Quantitave analysis of PET studies

The last frame of dynamic PET imaging (i.e. 55–60 min post-injection) was used to define regions of interest (ROIs) for quantitative analysis. Radioactivity concentration in a ROI was calculated with an automated system defining a 3 × 3 pixel ROI with maximum radioactivity within a larger user-defined ROI comprising the whole tumour (Minn et al, 1995). The relative standard deviation (s.d.) of the measured average radioactivity concentration in the maximum ROI was found to be less than 10%. The average of maximum ROIs in three consecutive planes was used in the final analysis, where only one representative tumour per patient having the highest radioactivity within the field-of-view was selected.

Tracer accumulation in a ROI at the end of the dynamic study was reported as the standardized uptake value (SUV), which is the radioactivity concentration in a ROI divided by the injected dose normalized to the patient's weight at a fixed time point (Woodard et al, 1975). The same formula was also applied for calculation of the SUV adjusted to the predicted value of lean body mass (Zasadny et al, 1993) and body surface area (BSA) (Olefsky et al, 1991). In addition, a graphical analysis of the tracer uptake was used as described earlier (Patlak et al, 1985). The slope of the linear plot obtained in the graphical analysis is equal to the utilization constant of [$F^{18}$]FDG (influx constant, $K_i$), which represents the fractional rate of tracer transport and phosphorylation per unit time in tissues with negligible reverse metabolism. In the current study, the last nine time points, representing the time from 15 to 60 min after injection, were used to determine the slope of the regression line. The [$F^{18}$]FDG influx constant was multiplied by the average plasma glucose level during imaging to obtain a metabolic index for [$F^{18}$]FDG utilization (the regional metabolic rate [$rMR_{FDG}$], μmol 100 $g^{-1}$ $min^{-1}$). The value for lumped constant was set to unity with awareness of difficulties in measuring true glucose utilization rates in heterogenous tissues (Spence et al, 1998). All SUV and $rMR_{FDG}$ values were calculated blinded without any knowledge of the clinical or other data.

In those four patients whose heart was within the field-of-view, large ROIs were drawn covering the whole myocardium (Knuuti et al, 1992). The fractional utilization constants of [$F^{18}$]FDG ($K_i$) and rates of regional myocardial glucose utilization (rMGU) were calculated as explained above. The value of 0.67 for lumped constant was used in the myocardial analysis (Ratib et al, 1982).

### Blood samples

Plasma glucose was determined in duplicate by the glucose oxidase method (Kadish et al, 1968) using an Analox GM7 (Analox Instruments, Copenhagen, Denmark) glucose analyser. Serum insulin was measured by radioimmunoassay (Kuzuya et al, 1977), serum FFA level with a fluorometric methods (Miles et al, 1983) and lactate by enzymatic analysis (Marbach et al, 1967).

### Histologic classification

Histology of lymphomas was classified according to the Working Formulation scheme (The Non-Hodgkin's Lymphoma Pathologic Classification Project, 1982) and the updated Kiel classification (Stansfeld et al, 1988). Four of the patients had low-grade, three intermediate-grade and two had high grade lymphoma. Three patients had Hodgkin's disease.

### Statistical analysis

The results are expressed as median and range, or mean ± s.d. where appropriate. Paired samples were compared by paired-comparisons *t*-test. A *P*-value of less than 0.05 was considered to be significant.

### RESULTS

### Metabolic characteristics during PET studies

Plasma glucose, serum insulin and lactate concentrations were similar during the two PET study approaches. Plasma glucose values were 5.1 ± 0.7 mmol $l^{-1}$ (range 3.8–6.7) at baseline and 4.8 ± 0.5 mmol $l^{-1}$ (range 3.6–5.8) after acipimox administration. The corresponding insulin concentrations were 5 ± 2 mU $l^{-1}$ (range 3–9) and 4 ± 1 mU $l^{-1}$ (range 3–7) respectively, and lactate concentrations 1.3 ± 0.3 mmol $l^{-1}$ (range 0.6–5.7) and 1.4 ± 1.8 mmol $l^{-1}$ (range 0.6–7.6) respectively. As expected, serum FFA concentrations were significantly reduced after acipimox administration. The serum FFA values were 0.92 ± 0.42 mmol $l^{-1}$ (range 0.55–1.88) at baseline and 0.26 ± 0.31 mmol $l^{-1}$ (range 0.03–1.12) after acipimox administration ($P$ = 0.0003, Figure 2).

### FDG uptake in tumour

The size and location of tumours are shown in Table 2. All tumours could be easily detected at PET because of increased

© Cancer Research Campaign 1999

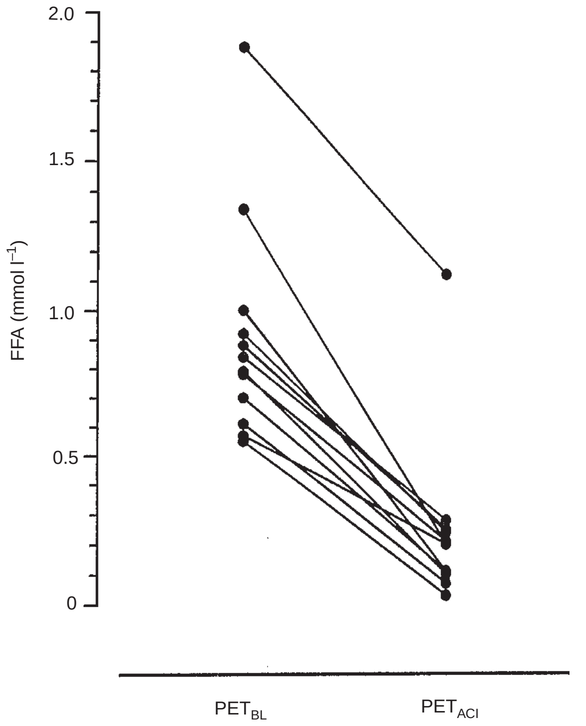

**Figure 2** The serum FFA concentration at baseline ($PET_{BL}$) and after acipimox administration ($PET_{ACI}$)

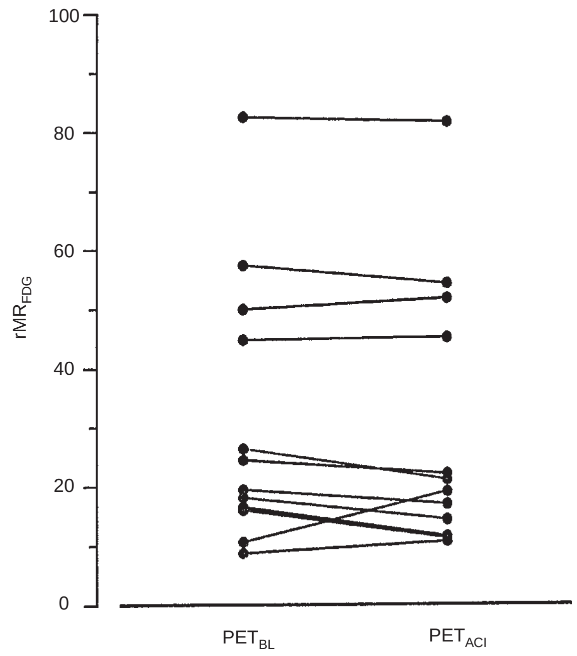

**Figure 3** Metabolic rate for [F[18]]FDG ($rMR_{FDG}$) at baseline ($PET_{BL}$) and after administration of acipimox ($PET_{ACI}$) in 12 patients with lymphoma uptake of [F[18]]FDG. The tumour visualization was never affected by administration of acipimox. Median $rMR_{FDG}$ of total of 12 involved lymph nodes was 21.9 μmol 100 $g^{-1}$ $min^{-1}$ (range 8.7–82.5) at baseline and 20.1 μmol 100 $g^{-1}$ $min^{-1}$ (range 10.7–81.7) after administration of acipimox. The respective figures for median SUV were 7.8 (range 3.6–18.6) and 6.0 (range 4.1–20.2) (Table 2). The differences in $rMR_{FDG}$ and SUV values between the two approaches were not statistically significant (Figure 3). Futhermore, no differences were seen when the tumour SUV was adjusted according to the predicted lean body mass or BSA (data not shown). There was a high correlation between $rMR_{FDG}$ and SUV both at baseline ($r^2 = 0.89$, $P < 0.0001$) and after acipimox treatment ($r^2 = 0.82$, $P < 0.0001$).

The trend for higher SUV and $rMR_{FDG}$ under both PET studies was seen in higher malignancy grades. The median SUV was 5.5 (range 4.4–6.1) in the low-grade, 7.8 (range 3.6–17.6) in the intermediate-grade and 18.4 (range 16.5–20.2) in the high-grade lymphomas. The respective values for median $rMR_{FDG}$ were 16.1 (range 11.3–19.4), 23.3 (range 8.7–57.4) and 66.8 μmol 100 $g^{-1}$ $min^{-1}$ (range 49.9–82.5). Indeed, the grade dependency of [F[18]]FDG uptake in lymphoma has been confirmed previously (Lapela et al, 1995). There was no trend of patients with a history

**Table 2** Tumour location, size and FDG uptake of PET studies

| Patient | Tumour location | Lesion size (cm) | $SUV_{BL}$ | $SUV_{ACI}$ | $rMR_{BL}$ | $rMR_{ACI}$ |
|---|---|---|---|---|---|---|
| 1 | Axilla | 4 × 3 | 5.3 | 4.4 | 16.2 | 11.3 |
| 2[a] | Retroperitoneum | 13 × 7 × 17 | 8.6 | 7.0 | 24.4 | 22.1 |
| 3 | Neck | 3 × 3.5 | 6.9 | 5.5 | 18.1 | 14.4 |
| 4 | Axilla | 2 × 2 × 3 | 6.1 | 5.5 | 19.4 | 17.0 |
| 5 | Mediastinum | 4 × 4.5 × 5 | 10.3 | 6.0 | 26.3 | 21.1 |
| 6[a] | Mediastinum | 8 × 9 × 13 | 13.2 | 14.7 | 44.8 | 45.2 |
| 7[b] | Gall-bladder | 7 × 7 × 8 | 18.6 | 16.5 | 82.5 | 81.7 |
| 8 | Retroperitoneum | 2 × 3 × 3.5 | 5.9 | 5.4 | 16.0 | 11.3 |
| 9[a] | Mediastinum | 2.5 × 5 × 2 | 5.0 | 6.0 | 10.6 | 19.1 |
| 10 | Neck | 5 × 3 | 18.2 | 20.2 | 49.9 | 51.8 |
| 11[a] | Axilla | 2 × 3.5 × 7 | 3.6 | 4.1 | 8.7 | 10.7 |
| 12 | Neck | 8 × 10 | 14.8 | 17.6 | 57.4 | 54.3 |
| | | Median | 7.8 | 6.0 | 21.9 | 20.1 |

Lesion size: three perpendicular diameters were measured from CT scans. SUV: the standardized uptake value. rMR: the regional metabolic rate μmol 100 $mg^{-1}$ $min^{-1}$. $SUV_{BL}$ and $rMR_{BL}$: values under high fasting FFA concentrations. $SUV_{ACI}$ and $rMR_{ACI}$: values under pharmacologically decreased FFA concentrations. [a]Patients who presented with a history of weight loss. [b] Patient with hypothyroidism.

© Cancer Research Campaign 1999

of recent weight loss to have a higher tumour FDG uptake than those without B-symptoms.

### [$F^{18}$]FDG uptake in the heart

In those patients whose heart was located within the field-of-view the myocardial glucose uptake was 11 ± 12 μmol 100 $g^{-1}$ $min^{-1}$ (range 3.4–31.8) at baseline. After acipimox administration myocardial glucose uptake was significantly higher and averaged 50 ± 24 μmol 100 $g^{-1}$ $min^{-1}$ (range 14.0–77.4, $P < 0.05$).

### DISCUSSION

We used PET to study the relationship between serum FFA and glucose metabolism in malignant lymphoma. We employed a nicotinic acid derivative, acipimox to decrease typically high post-absorptive FFA concentrations and the metabolic response of tumour was measured by [$F^{18}$]FDG and PET. The results indicate that even a striking reduction in circulating FFAs has little influence on the rate of glucose uptake in tumour as assessed by [$F^{18}$]FDG PET. This suggests that, in contrast to normal peripheral tissues, glucose uptake and FFA metabolism are uncoupled in malignant lymphoma.

At whole body level, FFAs inhibit glucose uptake, especially glucose oxidation (Thiebaund et al, 1982). The interaction between glucose and FFA metabolism was first demonstrated by Randle et al (1963) in a perfused rat heart. Thereafter, the glucose–FFA cycle has been shown to be operational and important in the human heart (Nuutila et al, 1992; Knuuti et al, 1995) and skeletal muscle (Nuutila et al, 1994) as well as at whole body level (Thiebaund et al, 1982). Although several enzymes are involved in glycolysis, only a few key enzymes regulate the glucose flux. Phosphofructokinase is an enzyme that controls partially the selection of fuels. The activity of phosphofructokinase is inhibited by excess of citrate and adenosine 5′-triphosphate (ATP) produced by metabolism of FFA and lactate. This leads to accumulation of glucose 6-phosphate, which restrains further uptake and phosphorylation of glucose by allosteric inhibition of hexokinase (Randle et al, 1964). Conversely, when citrate and ATP levels are decreased, the inhibitory effects are reduced and the activity of phosphofructokinase increases, leading to increased glucose transport and phosphorylation. Glycolysis is also regulated by pyruvate dehydrogenase (PDH), which converts pyruvate to acetyl–CoA (coenzyme A) that enters the citrate cycle. When PDH is inhibited, the pyruvate is diverted to the production of lactate (Neely et al, 1974). In malignant tissue, the activities of the key glycolytic enzymes tend to be enhanced (Weber 1977).

While the function of the glucose–FFA cycle has been well-established in normal tissues and healthy subjects, the picture is totally different in cancer patients who show tumour-associated changes in host metabolism. The depletion of fatty acids from the apidose tissue and increased fasting lactate levels are commonly found in patients even with a small tumour burden (Tayek, 1992). In our patients, the median plasma concentration of both FFA and lactate was very close to the upper normal limit and some individual patients showed high values that possibly reflected increased lipolysis and reduced oxidative metabolism induced by tumour. This is in line with the high uptake of [$F^{18}$]FDG in tumour, which is thought to represent increased anaerobic glucose metabolism and lactate release of malignant tissues. However, since [$F^{18}$]FDG is not metabolized beyond the initial phosphorylation step, it is not possible to determinate whether changes in [$F^{18}$]FDG uptake are related to oxidative or non-oxidative glucose disposal. Experimental studies indicate that oxygen consumption in cancer cells is lower in comparison to their parent normal tissues and glucose consumption, in turn, is five- to tenfold higher, leading to excessive production of lactate (Lehninger et al, 1975). We hypothesize that, since tumours fail to effectively convert pyruvate to acetyl–CoA to enter the citrate cycle, there is also a general failure of tumours to use FFA to meet the needs of the apparently defective oxidative energy metabolism. The uptake of FFA is thus directed mainly to the phospholipid pool to provide constituents for cell membranes of the proliferating tumour and high energy phosphates are produced through glycolysis even in the presence of excessive FFA.

The high caloric value of fat renders it the preferred source of energy in catabolic states such as malignancy and sepsis. Increased lipolytic activity is commonly seen in the serum of patients with advanced cancer, but a direct evidence of fatty acids as an important substrate for tumour energy metabolism has not been demonstrated in cancer patients (Toomey et al, 1995). Increased lipid mobilization and oxidation of fatty acids reflect redistribution of energy resources between tumour and host, but it is likely that degradation of lipids through oxidation does not involve tumour tissue which typically is more or less hypoxic. Although the anti-lipolytic drug acipimox has not been used to treat cachexia in patients (Obeid et al, 1997), we could demonstrate a significant drug-induced decrease in serum FFA without a change in tumour glucose metabolism. It is thus uncertain whether changes in supply of FFA have any effect on tumour growth which is fuelled by glycolysis, independent of other energy resources. Since low-serum FFA is not associated with increased rate of [$F^{18}$]FDG in tumour, we encourage further evaluation of anti-lipolytic drugs for treatment of cachexia and demonstrate the feasibility of PET for in vivo evaluation of metabolic effects in tumour and normal tissues. We underline that the results of the present study do not preclude the significance of fatty acids for the promotion of tumour proliferation or tumorigenesis (Nogushi et al, 1995).

In lymphoma, elevated accumulation of [$F^{18}$]FDG was first seen with a specially collimated γ-camera (Paul, 1987). The use of [$F^{18}$]FDG PET for detection of lymphoma has been established by several groups (Conti et al, 1996) and association between malignancy grade and rate of [$F^{18}$]FDG uptake has been suggested in our recent study (Lapela et al, 1995). We suggested earlier that oncologic patients should be imaged with [$F^{18}$]FDG in the fasting state, and the present study lends further support for this principle since [$F^{18}$]FDG signal in tumour is not influenced by competing substrates such as FFA. Although the relationship between [$F^{18}$]FDG uptake and true glucose metabolism in heterogenous tissues is obscured it does not invalidate current findings which are based on pairwise comparisons of the same tumours studied twice within 1 week. Because of the very small differences in the measured [$F^{18}$]FDG uptake in tumour, it is unlikely that a larger number of patients would have changed the results.

In conclusion, we have used [$F^{18}$]FDG PET to demonstrate the lack of an operational glucose–FFA cycle in tumour in patients with lymphoma. In neoplastic tissue, supply of FFA does not regulate use of glucose as a substrate for tumour growth. This supports earlier observations that indicate that tumours favour glycolysis to meet the needs of their energy metabolism due to limited oxidation of intracellular metabolites of glucose and FFA. We encourage the development of anti-lipolytic drugs for treatment of cachexia

© Cancer Research Campaign 1999

(Obeid et al, 1997), although no direct effect in terms of decreased use of glucose could be seen in lymphomatous tissue.

### ACKNOWLEDGEMENTS

The authors thank Professors Eeva Nordman and Uno Wegelius for valuable support. We also acknowledge assistance by Kerttu Irjala, MD and Olli Peltola, MSc for laboratory analysis of FFA and the staffs of the Turku PET Centre and Department of Oncology and Radiotherapy for their cooperation. Financial support was provided by the Finnish Cancer Society and Ida Montin Foundation.

### REFERENCES

Carbone PP, Kaplan HS, Musshoff K, Smithers DW and Tubiana M (1971) Report of the Committee on Hodgkin's Disease Staging Classification. *Cancer Res* **31**:1860–1861

Cederbaum AI and Rubin E (1976) Fatty acid oxidation, substrate shuttles, and activity of the citric acid cycle in hepatocellular carcinomas of varying differentation. *Cancer Res* **36**: 2980–2987

Conti PS, Lilien DL, Hawley K, Keppler J, Grafton ST and Bading JR (1996) PET and [$^{18}$F]-FDG in oncology: a clinical update. *Nucl Med Biol* **23**: 717–735

Gullino PM, Grantham FH and Courtney AH (1967) Glucose consumption by transplanted tumors in vivo. *Cancer Res* **27**: 1031–1040

Hamacher K, Coenen HH and Stöcklin G (1986) Efficient stereospecific synthesis of no-carrier-added 2-[$^{18}$F]-fluoro-2-deoxy-D-glucose using aminopolyether supported nucleophilic substitution. *J Nucl Med* **27**: 235–238

Kadish AH, Little RL and Strenberg JC (1968) A new and rapid method for the determination of glucose by measurement of rate of oxygen consumption. *Clin Chem* **14**: 116–131.

Kern KA and Norton JA (1988) Cancer cachexia. *J Parent Enteral Nutr* **12**: 286–298.

Knuuti MJ, Nuutila P, Ruotsalainen U, Saraste M, Härkönen R, Ahonen A, Hartiala J, Teräs M, Haaparanta M, Wegelius U, Haapanen A and Voipio-Pulkki L-M (1992) Euglycemic hyperinsulinemic clamp and oral glucose load in stimulating myocardial glucose utilization during positron emission tomography. *J Nucl Med* **33**: 1255–1262

Knuuti MJ, Mäki M, Yki-Järvinen H, Voipio-Pulkki LM, Härkönen R, Haaparanta M and Nuutila P (1995) The effect of insulin and FFA on myocardial glucose uptake. *J Mol Cell Cardiol* **27**: 1359–1367

Kuzuya H, Blix BM, Horwitz DL, Steiner DF and Rubenstein A (1977) Determination of free and total insulin and C-peptide in insulin-treated diabetics. *Diabetes* **26**: 22–29

Lapela M, Leskinen S, Minn H, Lidholm P, Klemi P, Söderström K-O, Bergman J, Haaparanta M, Ruotsalainen U, Solin O and Joensuu H (1995) Increased glucose metabolism in untreated non-Hodgkin's lumphoma: A study with positron emission tomography and fluorine-18-fluorodeoxyglucose. *Blood* **9**: 3522–3527

Laverazzi M, Milanesi E, Oggioni E and Pamparana F (1989) Results of a phase IV study carried out with acipimox in type II diabetic patients with concomitant hyperlipidemia. *J Int Med Res* **17**: 373–380

Lehninger AL (1975) Organ interrelationships in the metabolism of mammals. In *Biochemistry*, pp. 829–852. Worth: New York

Marbach E and Weil M (1967) Rapid enzymatic measurement of blood lactate and pyruvate. *Clin Chem* **13**: 314–325

Miles J, Glasscock R, Aikens J, Gerich J and Haymond M (1983) A microfluorometric method for determination of free fatty acids in plasma. *J Lipid Res* **24**: 96–99

Minn H, Nuutila P, Lindholm P, Ruotsalainen U, Bergman J, Teräs M and Knuuti J (1994) In vivo effects of insulin on tumor and skeletal muscle glucose metabolism in patients with lymphoma. *Cancer* **73**: 1490–1498

Minn H, Zasadny KR, Quint LE and Wahl RL (1995) Lung cancer: reproducibility of quantitative measurements for evaluating 2-(F-18)-fluoro-2-deoxy-*D*-glucose uptake in PET. *Radiology* **196**: 167–173

Mulligan HD, Beck SA and Tislade MJ (1992) Lipid metabolism in cancer cachexia. *Br J Cancer* **66**: 57–61

Nariai T, DeGeorge JJ, Greig NH, Genka S, Rapoport SI and Pyrdon AD (1994) Differences in rates of incorporation of intravenously injected radiolabeled fatty acids into phospholipids of intacerebrally implanted tumor and brain in awake rats. *Clin Exp Metastasis* **12**: 213–225

Neely JR and Morgan HE (1974) Relationship between carbohydrate and lipid metabolism and the energy balance of heart muscle. *Ann Rev Physiol* **36**: 413–459

Nogushi M, Rose DP, Earashi M and Miayazaki I (1995) The role of fatty acids and eicosanoid synthesis inhibitors in breast carcinoma. *Oncology* **52**: 265–271

Norton JA, Burt ME and Brennan MF (1980) In vivo utilization of substrate by human sarcoma-bearing limbs. *Cancer* **45**: 2934–2939

Nuutila P, Koivisto VA, Knuuti J, Ruotsalainen U, Teräs M, Haaparanta M, Bergman J, Solin O, Voipio-Pulkki L-M, Wegelius U and Yki-Järvinen H (1992) Glucose-free fatty acid cycle operates in human heart and skeletal muscle in vivo. *J Clin Invest* **89**: 1767–1774

Nuutila P, Knuuti MJ, Raitakari M, Ruotsalainen U, Teräs M, Voipio-Pulkki LM, Haaparanta M, Solin O, Wegelius U and Yki-Järvinen H (1994) Effect of antilipolysis on heart and skeletal muscle glucose uptake in overnight fasted humans. *Am J Physiol* **267**: E941–946

Obeid OA and Emery PW (1997) Effect of acute acipimox administration on the rates of lipid and glygogen synthesis in cachectic tumor-bearing rats. *Nutr Cancer* **28(1)**: 100–106

Olefsky JM (1991) Obesity. In *Harrison's Principles of Internal Medicine*, Vol 1, Wilson JD, Barunwald E, Isselbacher KJ, Petersdorf RG, Martin JB, Fauci AS, Root RK (eds), p. 411. McGrawHill: New York

Patlak CS and Balsberg RG (1985) Graphical evaluation of blood-to-brain transfer constants from multiple-time uptake data. Generalizations. *J Cereb Blood Flow Metab* **5**: 584–590

Paul R (1987) Comparison of fluorine-18-2-fluorodeoxyglucose and gallium-67 citrate imaging for detection of lymphoma. *J Nucl Med* **28**: 288

Phelps ME, Huang SC, Hoffman EJ, Selin C, Sokoloff L and Kuhl DE (1979) Tomographic measurement of local cerebral glucose metabolic rate in humans with (F-18)2-fluoro-2-deoxy-*D*-glucose: validation of method. *Ann Neurol* **6**: 371

Randle PJ, Garland PB, Hales CN and Newsholme EA (1963) The glucose fatty acid cycle: its role in insulin sensitivity and the metabolic disturbances of diabetes mellitus. *Lancet* **1**: 785–789

Randle PJ, Newsholme EA and Garland PB (1964) Regulation of glucose uptake by muscle. Effects of fatty acids, ketone bodies and pyruvate, and alloxan, diabetes and starvation, on the uptake and metabolism fate of glucose in rat heart and diaphragm muscles. *Biochem J* **93**: 652–665

Ratib O, Phelps ME, Huang S-C, Henze E, Selin CE and Schelbert HR (1982) Positron tomography with deoxyglucose for estimating local myocardial glucose metabolism. *J Nucleic Med* **23**: 577–586

Shipp MA and Harrington DP (1993) Chairpersons in the international non-Hodgkin's lymphoma prognostic factors project: a predictive model for aggressive lymphoma. *N Engl J Med* **329**: 987

Spence AM, Muzi M, Graham MM, O'Sullivan F, Krohn KA, Link JM, Lewellen TK, Lewellen B, Freeman S, Berger M and Ojemann GA (1998) Glucose metabolism in human malignant gliomas measured quantitatively with PET, 1-[C-11]glucose and FDG: analysis of the FDG lumped constant. *J Nucl Med* **39**: 440–448

Spinks TJ, Jones T, Gilardi MC and Heather JD (1988) Physical performance of the latest generation of commercial positron scanners. *IEEE Trans Nucl Sci* **35**: 721–725

Stansfeld AG, Diebold J, Noel H, Kapanci Y, Rilke F, Kelenyi G, Sundström C, Lennert K, van Unnik JAM, Mioduszewska O and Wright DM (1988) Updated Kiel classification for lymphomas. *Lancet* **1**: 292–293

Tayek JA (1992) A review of cancer cachexia and abnormal glucose metabolism in humans with cancer. *J Am Coll Nutr* **11**: 445–456

The Non-Hodgkin's Lymphoma Pathologic Classification Project (1982) National Cancer Institute sponsored study of classification of non-Hodgkin's lymphomas: summary and description of working formulation for clinical usage. *Cancer* **49**: 2112

Thiebaund D, DeFronzo RA, Jacot E, Golay A, Acheson K, Maeder E, Jequir E and Felber JB (1982) Effect of long-chain triglyceride infusion on glucose metabolism in man. *Metab Clin Exp* **31**: 1128–1136

Toomey D, Redmond P and Bouchier-Hayes D (1995) Mechanisms mediating cancer cachexia. *Cancer* **76**: 2418–2426

Wahl RL, Zasadny K, Helvie M, Hutschins GD, Weber B and Cody R (1993) Metabolic monitoring of breast cancer chemohormonotherapy using positron emission tomography: initial evaluation. *J Clin Oncol* **11**: 2101–2111

Warburg O (1930) *The Metabolism of Tumours*. Constable: London

Weber G (1977) Enzymology of cancer cells (Part II). *N Engl J Med* **296**: 541–551

Woodard HO, Bigler RE and Freed B (1975) Expression of tissue isotope distribution. *J Nucl Med* **16**: 958–959

Zasadny K and Wahl RL (1993) Standardized uptake values of normal tissues at PET with 2-[fluorine-18]-fluoro-2-deoxy-D-glucose: variations with body weight and a method for correction. *Radiology* **189**: 847–850

 © Cancer Research Campaign 1999
